# Validation of the Health Consciousness Scale among the Czech Population

**DOI:** 10.3390/healthcare11111628

**Published:** 2023-06-02

**Authors:** Jiri Remr

**Affiliations:** INESAN (Institute for Evaluations and Social Analyses), Sokolovská 351/25, 18600 Prague, Czech Republic; jiri.remr@inesan.eu

**Keywords:** health consciousness, psychometric validation, cross-sectional survey, COVID-19

## Abstract

The COVID-19 pandemic has demonstrated the importance of maintaining good health. It became has become apparent that health consciousness is a crucial factor in promoting healthy lifestyles, disease prevention, and the overall well-being of individuals. A higher level of health consciousness is associated with healthy habits, better adherence to medical recommendations, and a higher quality of life. Therefore, health consciousness is a critical construct in health care that reflects the degree to which individuals care about their health. This study, which is based on a representative sample of the adult population (n = 1372), aims to validate the Health Consciousness Scale (HCS) to assess its reliability and validity, and evaluate the factor structure of the translated version of the scale in the Czech language. The validation of the HCS in the Czech context is a significant step forward and provides useful information for healthcare professionals, policymakers, and researchers. The findings of this study contribute to the understanding of health consciousness in the Czech population and provide unique information for the development and evaluation of health interventions aimed at promoting healthy behaviors and attitudes.

## 1. Introduction

In the context of the COVID-19 pandemic, health and health-related information have been brought to the forefront of the public consciousness. The pandemic has had far-reaching consequences that have affected many aspects of life, including physical and mental health [1]. The provision of regular information on the nature of the disease, its symptoms, and incidence rate has been crucial in stimulating and increasing attention towards personal health. The media has played a vital role in disseminating highly detailed information on the possible modes of infection, explaining the ways in which the disease can spread, the associated symptoms, and measures to reduce the risk of infection [2]. As a result, a significant proportion of the population has become more interested in their own health and the well-being of their loved ones [3].

The COVID-19 pandemic has brought the importance of health to the forefront of public discourse. However, the awareness of personal health and monitoring it appropriately is essential, not only during a pandemic but also under regular circumstances. This is because adequate attention to one’s health, and identifying changes in one’s health status, can prevent the onset of serious diseases, enable early intervention and treatment, and improve the prospects for recovery [4]. It can also lead to healthier lifestyles, thereby enhancing overall quality of life [5]. Several experts, e.g., [6,7,8], have emphasized the importance of individual health consciousness and proper prevention. The COVID-19 pandemic has made people acutely aware of the fragility of life and the value of good health [9].

Health consciousness is a critical construct in health care and has been studied extensively. The subjective characteristics of an individual motivates them to be aware of and involved in their own health. As mentioned by Gould [10], health consciousness is the degree to which an individual is concerned about their health. It is a psychological construct that has been shown to influence health-related and preventive behaviors to act towards maintaining good health. Gould [11] emphasized that health consciousness is closely related to one’s perception of their health status and, in this respect, he described health consciousness as self-consciousness regarding one’s own health.

In light of the great importance of health awareness, the Health Consciousness Scale (HCS) is a tool that can be used to measure an individual’s consciousness and understanding of health-related issues. The HCS is a self-report instrument that assesses an individual’s level of attention to health and health behaviors; it measures the level of knowledge about the body, the importance of health, and the impact of lifestyle on health [10]. For completeness, it should be mentioned that the basis of HCS was a total of four sub-dimensions, namely health self-consciousness, health involvement, health alertness, and health self-monitoring. Health self-consciousness explains that individuals who have greater health consciousness tend to show more concern when they are in proximity to health risks. They are more mindful of their health and tend to engage in health-related behaviors. Health self-consciousness directly influences a person’s health care behaviors. The second dimension (health involvement) refers to the search for and use of health information. Health-conscious individuals tend to be more concerned about their health, which includes seeking and using health information. Health alertness is the third dimension, which involves taking responsibility for one’s own health. Individuals with higher levels of health consciousness tend to be more mindful of their physical condition, stress management, and nutrition. The final dimension is health self-monitoring, which refers to the intensity with which individuals value a healthy state. In addition, health self-efficacy is a key component of health consciousness, indicating an individual’s confidence in their ability to maintain good health. These four dimensions were at the birth of HCS and, according to Gould, can be used independently. Indeed, their convergent validity was examined with this in mind. However, in addition to the initial first-order factor model, Gould also conducted a second-order confirmatory factor analysis to examine whether all four subscales could be combined, and whether a common scale could be used. This was indeed confirmed, and so the whole scale can be used as a single measure [10]. In view of these results, and considering the way other authors have used the scale [12,13], this study treats the whole scale as a unidimensional construct.

Studies have shown that higher levels of health consciousness positively correlate with healthy lifestyles, adherence to medical recommendations, and higher levels of prevention and related self-care [9,14,15]. Additionally, individuals with higher levels of health consciousness tend to have a higher perceived quality of life and lower levels of health-related anxiety and worry [5,16]. They are also more likely to take responsibility for their own health and have higher levels of self-efficacy [11].

Health consciousness has gained greater importance due to the ongoing COVID-19 pandemic [17]. Preventive behaviors, such as physical activity and adherence to health guidelines, have been important in protection against the virus [18]. Health-conscious individuals were more likely to understand their own health, paid attention to health problems, and took the necessary measures to ensure their well-being [19]. However, the HCS has not yet been validated in the Czech context, nor has it been translated or utilized in relevant studies produced by researchers in this country.

Therefore, the primary objective of this study was to translate the HCS into the Czech language and establish its psychometric properties in a general adult population in Czechia. The objectives of this study are to examine the factor structure of the Health Consciousness Scale developed by Gould, validate the translated version of the scale in the Czech language, and assess its reliability and validity. The study aimed to create a valid and reliable tool that could be used to measure health consciousness in the Czech population. Validation of the HCS enabled researchers to use the scale easily and assess the level of health consciousness in specific sub-populations. Additionally, the baseline values of each dimension and indicators were established, serving as reference values or benchmarks. This information could be useful to healthcare professionals, policymakers, and researchers, as it provides insight into the health consciousness of the population, which can in turn aid in the development of health promotion strategies and public health policies.

## 2. Materials and Methods

### 2.1. Participants and Procedure

The objective of this survey was to gather valuable insights from the general population of Czechia, specifically targeting individuals aged 15 to 74. To achieve a representative sample, a multistage random sampling technique was employed, utilizing address-based sampling. Due to limited access to an appropriate sampling frame or resident list, primary sampling units were selected, and addresses within each unit were identified, from which households were then sampled. Subsequently, trained interviewers visited pre-selected addresses and identified the potential respondents using the Kish table [20].

To represent the theoretical population, 174 primary sampling units were carefully identified; for each of these primary sampling units, a maximum of 20 addresses were assigned. A total of 2657 households were contacted during the data collection stage of the research, and ultimately, 1423 face-to-face interviews were successfully conducted, yielding a response rate of 53.2%. Prior to conducting each interview, informed consent was obtained from the participants. To ensure the confidentiality of respondents, all responses were anonymized and presented only in an aggregated format, thereby guaranteeing that the direct and indirect identification of any individual is not possible.

The fieldwork for this survey was carried out in June 2020, shortly after the so called first wave of the COVID-19 outbreak. On average, each interview lasted approximately 20 min. To maintain high standards of quality and ethical conduct, 35% of the interviews were subject to the check-backs when the interviews were carried out, as well as examining the performance of the interviewers with respect to their compliance with relevant guidelines. As a result of some incomplete interviews, the final dataset that was used for the analysis contained 1372 cases; such sample size was deemed sufficient to validate the scale. Additionally, the sample was carefully structured to ensure representation across gender, age, and size of the settlement, as depicted in Table 1.

Before the data collection, the research tool (i.e., HCS) was translated into the Czech language. The process of translation followed the recommendation of Sousa and Rojjanasrirat [21] and Yu, Lee, and Woo [22]. Therefore, two parallel translations were performed by two independent translators, who translated the English version of the HCS into the Czech language. Then, the two translations of all nine items were compared and the differences were resolved. In this way, an integrated form of the Czech translation of the scale was created. After that, the third translator performed back-translation to confirm the equivalence of the English and Czech versions. The alternatives (agreement with statements) have also been taken from the original scale. The translated scale was then pilot-tested on a sample of 27 respondents recruited from the target population. However, only minor changes in the wording of one item were made because there were no issues concerning understandability or vagueness. Think-aloud interviews were conducted for this purpose [23]. The Czech version of the scale is in Table A1 in Appendix A.

### 2.2. Measures

#### 2.2.1. Health Consciousness Scale (HCS)

As it is mentioned above, Gould developed the Health Consciousness Scale (HCS) [10] to measure respondents’ attitudes and perceptions towards their own health. The HCS comprises nine items that are assessed on a five-point, Likert-type scale ranging from 1 = “not at all reflecting my situation” to 5 = “fully reflecting my situation”. The total score ranges from 9 to 45 with a higher score indicating greater health consciousness. Numerous studies, e.g., [24,25,26], have repeatedly used the scale and examined its reliability and validity.

#### 2.2.2. Direct Stimuli

Given that health consciousness is reflected in the content of an individual’s communication with others, and is also related to the effort people make to search for information related to their health, relevant indicators of individuals’ attempt to seek health information were also examined. Specific behavioral patterns were identified through simple direct questions with respondents specifying subjective frequency using six-point ordinal variables, where 6 = very often, 5 = often, 4 = sometimes 3 = seldom, 2 = exceptionally, 1 = never. The questions focused on sharing health information with the immediate social environment, as well as actively seeking health-relevant information on food packaging, in magazines and books, and on the Internet. Exact wording of these direct stimuli is obvious from Table 2. Similar questions were used by Gould in the process of HCS validation [10]. The underlying hypothesis here is that individuals with higher scores of health consciousness will report higher frequency of these behavioral patterns.

### 2.3. Data Analysis

In order to assess the content validity of the translated Health Consciousness Scale, rigorous adherence was given to prevailing scientific guidelines [27]. To provide comprehensive information about the sample and respondents involved in the study, a range of descriptive statistics were computed, including measures such as mean (M), standard deviation (SD), skewness, and kurtosis. To explore and test differences among the provided variables and indicators, Pearson’s correlation analyses and analyses of variance (ANOVA) were carried out. The internal consistency of the scale was assessed by Cronbach’s alpha coefficient [28,29,30].

To ensure the validity of the scale, an exploratory factor analysis with principal components estimation was conducted [31], followed by confirmatory factor analysis (CFA) employing the maximum likelihood estimation method. In this respect, the total sample was split into two halves by a rigorous, randomization procedure, assuring the equivalence of both subsamples. Consequently, exploratory-factor analysis was conducted on the first subsample, whereas the other subsample was used for confirmatory-factor analysis. Listwise deletion of missing cases was employed for the analyses of the HCS. However, it is important to note that other variables, such as the direct stimuli, may have varying numbers of valid cases. All statistical analyses were performed using IBM SPSS ver. 27 (IBM Corp., Armonk, NY, USA), except for confirmatory-factor analysis, which was conducted in AMOS 24.0

## 3. Results

### 3.1. Univariate Statistics

Table 3 shows a similar pattern of the standard deviations for all individual items; no item has standard deviation that is significantly different from the others. In this study, the HCS has a mean of 24.14; standard deviation is 8.529 and the median is 25. For comparison, the mean of the original scale reported by Gould [10] was 20.01 and the standard deviation was 8.53.

In this study, the scale produced a skewness of −0.075, and kurtosis equaled −0.664. Table 3 shows also both statistics for all nine items. Skewness and kurtosis were within the range of −1.5 to +1.5, so the scale might be considered to be normally distributed [32]. Floor effect was 7.9% and ceiling effect was 0.5%; these values were acceptable according to Cain et al. [33], who recommended that these values not exceed 50%.

### 3.2. Uni-Dimensionality and Internal Consistency

To assess the scale’s uni-dimensionality, an exploratory factor analysis was conducted. The results indicated that the scale was uni-dimensional, as only one factor with an eigenvalue > 1 was extracted using principal components analysis, which accounted for 70.1% of the total variance. According to Pett et al. [34], higher values suggested stronger item contributions to the factor’s explanation, and factor scores greater than 0.8 demonstrated that the individual items contributed substantially to explaining the overall factor. Based on Table 4, which shows the item loads on the first extracted factor and communalities, it is obvious that eight items had factor scores exceeding 0.8, while the remaining one had a score that came close to this threshold; its value was 0.772 (“I am very self-conscious about my health”), thus indicating a slightly weaker contribution.

The obtained findings provide support for the hypothesis proposing a uni-dimensional structure of health consciousness, as measured by the proposed scale. Notably, it is worth mentioning that the Kaiser–Meyer–Olkin (KMO) measure of sampling adequacy yielded a high value of 0.944, indicating that the data was highly suitable for factor analysis. Additionally, Bartlett’s Test of Sphericity revealed a significant result with χ^2^ = 9813.479 (df = 36, *p* < 0.001), suggesting that the correlation matrix was not an identity matrix. This implies that the variables were interrelated through underlying factor(s), further affirming the suitability of the data for factor analysis [35].

To evaluate the internal consistency of the scale, Cronbach’s alpha coefficient was computed, resulting in an impressive value of 0.947. This value exceeded the original scale’s reported reliability of 0.92, as indicated by Gould [10], signifying the inherent stability of the scale. In addition, item-total correlation as another indicator of internal consistency was used. According to Pakpour et al. [36], item-total correlations exceeding 0.4 were considered acceptable, and in this study, item-total correlations ranged from 0.733 to 0.829, exceeding the recommended threshold and indicating good internal consistency. These results provided support for the hypothesis that the items accurately reflected the intended construct.

### 3.3. Psychometric Performance of the Scale

**Figure 1:**
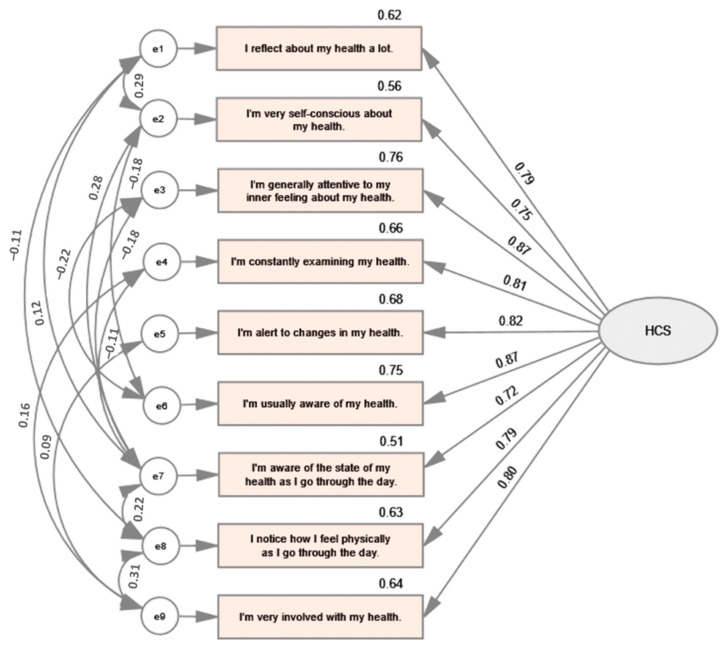
CFA of the Improved Model.

To assess the construct validity of the scale and its psychometric performance, a confirmatory factor analysis (CFA) was conducted using maximum likelihood estimation. The results revealed a significant chi-square value of 13.856 with one degree of freedom (*p* < 0.001). It is worth noting that obtaining a significant chi-square value is common when conducting CFA on the large samples, as aptly explained by Pituch and Stevens [37]. Therefore, it is essential to consider additional fit indices to obtain a more precise assessment of model fit, such as the comparative fit index (CFI), Tucker–Lewis index (TLI), and root–mean–square error of approximation (RMSEA). These indices can provide a more comprehensive and accurate representation of the scale’s fit to the underlying model. The tested model is presented in Figure 1.

To assess the model fit, a range of absolute and incremental indices were calculated. The resulting values, along with the recommended thresholds, are presented in Table 5. These fit indices can provide insight into the adequacy of the proposed model by evaluating how well it fits the observed data. During data analysis, the model was refined to account for errors representing unobserved variables that capture variance that is unaccounted for by the latent construct. The results of both the original and improved models are presented in Table 5.

The findings indicate that the proposed model exhibits a strong fit to the Czech data, substantiating the good psychometric performance of the Health Consciousness Scale. Notably, the key fit indices provided compelling support for this conclusion. The root–mean–square error of approximation (RMSEA) yielded a value of 0.028, which fell below the recommended threshold, suggesting a favorable fit between the hypothesized model and the collected data. Another absolute-fit index, the standardized root–mean–square residual (SRMR) that serves as an average difference between the observed covariance matrix and the implied covariance matrix based on the model, reflected an exceptional fit between the model and the data achieving the value of 0.010. Furthermore, the goodness of fit index (GFI), an incremental-fit index that quantifies the proportion of variance in the observed covariance matrix accounted for by the model, yielded an impressive value of 0.993, indicating an excellent fit. Additionally, the comparative-fit index (CFI), another incremental-fit index that compares the fit of the hypothesized model to a baseline model assuming no relationships among variables, obtained a value of 0.998, further bolstering the evidence of an excellent fit. Similarly, both the Tucker–Lewis index (TLI) and the normed-fit index (NFI), which are also incremental-fit indices comparing the fit of the hypothesized model to a baseline model, provided substantial support for an outstanding fit.

### 3.4. Convergent Validity

To establish the convergent validity of the Czech version of the HCS, the average variance extracted (AVE) and composite reliability (CR) were examined. AVE represented the average amount of variance captured by the indicators associated with the latent variable; a higher AVE value would have indicated stronger convergent validity, when the items in the construct were highly related to each other and were measuring the same underlying construct. In this respect, a value higher than 0.5 was recommended [41]. In this study, the Czech version of the HCS had an AVE of 0.70, indicating that, on average, 70% of the variance in the indicators was explained by the latent variable. These results suggested that the latent variable accurately represented the construct it was intended to measure. CR represented the degree to which the indicators of the latent variable were related to each other; a higher CR value would have indicated greater internal consistency, suggesting that the items are reliable and consistent in measuring the intended construct. For CR, a value above 0.7 was desirable [42]. The CR value for the Czech data was 0.96, indicating that the items of the latent variable were highly correlated with each other. These results provided further support for the convergent validity of the HCS, demonstrating the extent to which a construct was accurately measured and could be considered valid in the provided study.

### 3.5. Construct Validity

Establishing construct validity is a critical step in validating any research instrument or measure, as it provides evidence regarding the accuracy of the given scale measuring the intended construct [43]. Construct validity examines the extent to which the items are positively correlated with each other, indicating that they are measuring the same construct consistently [44]. In other words, each item on the scale should reflect the given concept, and the individual items should be correlated as predicted. The correlation matrix presented in Table 6 provides strong evidence for such construct validity, showing significant associations among all items, which indicates that they are measuring the same construct.

To expand the amount of evidence on construct validity and to show the usefulness of the HCS, the mean scores of the scale are compared for different subgroups of respondents. Table 7 shows how the HCS score differs among selected subgroups. In this respect, it is obvious that a higher level of HCS is observed among females and older respondents. Similarly, a higher HCS score can be observed among smokers (especially current smokers), and those who reported poor health status. There are also higher HCS scores among those who do not have an ideal weight (i.e., those who are either underweight or obese). On the other hand, there is no significant difference based on the population size of their place of residence.

The presented results suggest that the HCS is significantly correlated with direct measures reflecting respondents’ relevant attitudes and experiences concerning health behavior. Specifically, the one-way ANOVA results showed a significant effect for the statement “I talk about health issues with my relatives and friends”. (F(5, 9700) = 615.103, *p* < 0.001), “I read nutrition facts on the food packages”. (F(5, 25,164) = 1506.803, *p* < 0.001), “I search for health-related information in journals and books”. (F(5, 24,486) = 1445.426, *p* < 0.001), and “I am searching information on specific diseases on Internet”. (F(5, 23,166) = 1388.098, *p* < 0.001).

## 4. Discussion

The primary objective of this study was to comprehensively evaluate the psychometric characteristics of the Health Consciousness Scale (HCS) within Czech society. Multiple approaches were employed to assess the psychometric performance of the HCS, including internal consistency test principal components analysis, and confirmatory-factor analysis. The results indicated that the HCS performed well within the Czech context. Regarding distributional performance, the scale demonstrated acceptable skewness and kurtosis values falling within an acceptable range [32]. The item-total correlations exhibited values ranging from 0.733 to 0.829, surpassing the recommended threshold of 0.4, thereby meeting acceptable criteria [35]. Additionally, the scale displayed a unidimensional structure, high-internal consistency, satisfactory construct validity, and robust concurrent validity. Notably, the achieved standardized root–mean–square residual (SRMR) value of 0.012 felt within acceptable limits when compared to the recommended threshold of 0.080 [39]. Similarly, the root–mean–square error of approximation (RMSEA) value of 0.046 met the accepted threshold [38]. The comparative-fit index (CFI) and the Tucker–Lewis index (TLI) both exceeded the recommended threshold of 0.95, indicating a strong model fit [39]. Convergent validity, as indicated by the average variance extracted (AVE) and composite reliability (CR), also met the recommended thresholds of 0.5 and 0.6, respectively [41,42]. In summary, the HCS demonstrated excellent concurrent validity, exhibiting significant associations with all relevant direct variables used in the study.

Consistently with previous research findings, this study aligned with existing evidence demonstrating that lower levels of health consciousness were associated with reduced ability to communicate with healthcare professionals about illness symptoms, underutilization of preventive care, and overuse or overconsumption of healthcare services [45,46]. Furthermore, the research findings supported the association of health consciousness with age and gender, as revealed by previous studies [8,24,25,26]. Specifically, older individuals exhibited higher levels of health consciousness compared to younger respondents, while females displayed higher levels of health consciousness than males [47].

Although the primary focus of this study did not revolve around examining the association between health consciousness and health-related behaviors and compliance, it underscored the significance of further research in this area, particularly in the context of the ongoing COVID-19 pandemic. Further in-depth investigations are still needed to explore the detailed association between health consciousness and behaviors, as well as compliance with the imposed measures.

### Limitations

Given that the research conducted was a cross-sectional study, it is important to acknowledge that determining the direction of the association between health consciousness and self-reported attitudinal information is not feasible [48]. For instance, it is unclear whether increased pressure or poorer health status led to higher health consciousness, or whether it was higher health consciousness that resulted in increased pressure or lower perceived health status. Both explanations hold merit, but a different research design would be necessary to establish the direction of causality.

Another notable point in the study, is the reliance on self-reported data. While the study confirmed statistically significant associations between variables, the authors acknowledge the limitations of using self-reported data as opposed to independently confirmed objective measures [49]. This emphasizes the importance of using multiple data collection methods, including observational data to strengthen the validity of research findings.

The study was conducted during the COVID-19 pandemic, specifically three months after its onset, which was a phase characterized by a high level of information on the disease, its symptoms, and the measures to limit its spread. The author suggests that the attention to health issues during this period may have influenced the relevance of the data collected, potentially resulting in overestimation compared to non-pandemic periods. Therefore, it would be useful to compare the current HCS score with values from non-pandemic periods (once they are available) to obtain a comprehensive understanding of the impact of pandemics on health consciousness.

## 5. Conclusions

The study highlights the importance of behavior and compliance in managing public health, the need for multiple data collection methods, and the potential impact of the pandemic on the relevance of collected data. The HCS is an easy-to-administer tool that can provide valuable information on individuals’ health consciousness and could be used in future research studies.

The HCS has demonstrated good psychometric properties and can be a valuable tool for measuring health consciousness in Czech society. However, future research is needed to further investigate its association with health-related behaviors and compliance, and to compare its performance in different populations and contexts.

Research studies that investigate the determinants of individuals’ health status and behavior are essential in guiding policy and decision-making in the healthcare sector. The presented results contribute to the growing body of research on the impact of pandemics on populations by specifically focusing on health consciousness in the Czech setting.

## Figures and Tables

**Table 1:** Socio-demographic Characteristics of the Sample.

Variables		Theoretical Population *	Sample
Gender	Male	50.0%	50.0%
	Female	50.0%	50.0%
	Total	100.0%	100.0%
Age	15–24 years	11.5%	11.5%
	25–34 years	17.1%	17.1%
	35–44 years	21.2%	21.2%
	45–54 years	18.2%	18.2%
	55–64 years	16.3%	16.3%
	65–74 years	15.7%	15.7%
	Total	100.0%	100.0%
Size of settlement	Less than 1000 inhabitants	16.8%	16.8%
	1000 to 4999 inhabitants	22.6%	22.1%
	5000 to 19,999 inhabitants	18.2%	18.7%
	20,000 to 99,999 inhabitants	20.0%	20.3%
	100,000 inhabitants and more	22.3%	22.1%
	Total	100.0%	100.0%

* Data about the theoretical population comes from the Czech Statistical Office.

**Table 2:** Descriptive Statistics of the Direct Stimuli.

		6	5	4	3	2	1	N *
1	I talk about health issues with my relatives and friends.	23.2%	21.9%	20.8%	20.1%	10.4%	3.6%	1294
2	I read nutrition facts on the food packages.	11.9%	17.1%	20.1%	28.1%	14.7%	8.1%	1273
3	I search for health-related information in journals and books.	16.9%	12.7%	19.9%	25.4%	15.7%	9.4%	1294
4	I am searching information on specific diseases on Internet.	7.9%	10.2%	16.4%	25.9%	22.4%	17.2%	1233

* Note: N differs due to uneven number of missing cases.

**Table 3:** Descriptive Statistics of the Health Consciousness Scale (HCS) and Its Items.

	N	Mean	SD	Skewness	Kurtosis	Item-Total Correlation
I reflect about my health a lot.	1321	2.73	1.135	0.046	−0.800	0.796
I am very self-conscious about my health.	1321	2.47	1.092	0.261	−0.728	0.733
I am generally attentive to my inner feeling about my health.	1321	2.76	1.109	−0.012	−0.734	0.815
I am constantly examining my health.	1321	2.78	1.123	0.019	−0.753	0.804
I am alert to changes in my health.	1321	2.70	1.168	0.152	−0.841	0.799
I am usually aware of my health.	1321	2.89	1.134	−0.076	−0.761	0.829
I am aware of the state of my health as I go through the day.	1321	2.36	1.145	0.397	−0.798	0.741
I notice how I feel physically as I go through the day.	1321	2.70	1.134	0.076	−0.777	0.810
I am very involved with my health.	1321	2.76	1.132	0.107	−0.672	0.796
The whole HCS scale	1321	24.14	8.529	−0.075	−0.664	

**Table 4:** Exploratory Factor Analysis.

	N	F1	Communalities
I reflect about my health a lot.	660	0.854	0.73
I am very self-conscious about my health.	660	0.772	0.60
I am generally attentive to my inner feeling about my health.	660	0.859	0.74
I am constantly examining my health.	660	0.865	0.75
I am alert to changes in my health.	660	0.850	0.72
I am usually aware of my health.	660	0.886	0.78
I am aware of the state of my health as I go through the day.	660	0.807	0.65
I notice how I feel physically as I go through the day.	660	0.870	0.76
I am very involved with my health.	660	0.836	0.70

**Table 5:** Absolute and Incremental Indices.

Indices	Thresholds	Original Model	Improved Model
RMSEA (Root–mean–square Error of Approximation)	<0.07 [38]	0.121	0.028
SRMR (Standardized Root–mean–square Residual)	<0.08 [39]	0.036	0.010
GFI (Goodness of Fit Index); Adjusted GFI	>0.95 [40]	0.913	0.993
CFI (Comparative Fit Index)	>0.90 [39]	0.945	0.998
TLI (Tucker–Lewis Index)	>0.95 [39]	0.926	0.996
NFI (Normed fit index)	>0.95 [39]	0.944	0.995

**Table 6:** Correlational Matrix.

		1	2	3	4	5	6	7	8	9
1	I reflect about my health a lot.	1.000								
2	I am very self-conscious about my health.	0.635 **	1.000							
3	I am generally attentive to my inner feeling about my health.	0.642 **	0.579 **	1.000						
4	I am constantly examining my health.	0.616 **	0.521 **	0.647 **	1.000					
5	I am alert to changes in my health.	0.603 **	0.578 **	0.627 **	0.618 **	1.000				
6	I am usually aware of my health.	0.608 **	0.518 **	0.657 **	0.694 **	0.652 **	1.000			
7	I am aware of the state of my health as I go through the day.	0.574 **	0.626 **	0.534 **	0.516 **	0.561 **	0.543 **	1.000		
8	I notice how I feel physically as I go through the day.	0.563 **	0.539 **	0.649 **	0.592 **	0.589 **	0.653 **	0.627 **	1.000	
9	I am very involved with my health.	0.575 **	0.510 **	0.601 **	0.661 **	0.605 **	0.660 **	0.534 **	0.667 **	1.000

n = 1321; Kendall’s tau_b; ** = *p* < 0.01.

**Table 7:** Associations of HCS with Other Indicators.

	%	Mean	SD	F	*p*	Eta
Gender				16.157	*p* < 0.001	0.110
Male	50%	23.21	8.490			
Female	50%	25.09	8.471			
Age				26.426	*p* < 0.001	0.302
15–24 years	12%	20.38	8.682			
25–34 years	17%	22.75	8.476			
35–44 years	21%	22.36	7.860			
45–54 years	18%	23.95	8.203			
55–64 years	16%	26.54	7.604			
65–74 years	16%	28.53	8.233			
Size of Settlement				0.639	0.635	0.044
less than 1000 inhabitants	17%	23.59	10.137			
1000 to 4999 inhabitants	22%	24.50	8.217			
5000 to 19,999 inhabitants	19%	23.87	8.849			
20,000 to 99,999 inhabitants	20%	24.60	8.179			
100,000 inhabitants and more	22%	23.99	7.575			
BMI (body mass index) *				5.981	*p* < 0.001	0.118
underweight (less than 20.0)	5%	23.93	9.487			
ideal weight (20.00–24.9)	39%	23.03	8.405			
obesity (25.0–29.0)	43%	24.72	8.327			
heavy obesity (30.0 or more)	14%	25.81	8.909			
Smoking habits				12.054	*p* < 0.001	0.134
current smokers	37%	26.35	1.808			
past smokers	17%	24.85	1.912			
never smoked	46%	22.97	2.234			
Self-reported health status				222.100	*p* < 0.001	0.504
poor	13%	31.86	6.509			
quite good	43%	26.45	6.883			
good	44%	19.70	8.041			

* BMI was computed with the use of self-reported data on gender, age, height, and weight.

## Data Availability

The data used to support the findings of this study will be available from the corresponding author upon reasonable request.

## Appendix A

Table A1 in the Appendix A compares the original English version of HCS and its translation into Czech.

**Table A1:** Comparison of the English and Czech versions of the HCS.

English		Czech
1	I reflect about my health a lot.	Hodně přemýšlím o svém zdravotním stavu.
2	I am very self-conscious about my health.	Jsem velmi úzkostlivý/á ohledně mého zdraví.
3	I am generally attentive to my inner feeling about my health.	Věnuji pozornost svým vnitřním pocitům, které se týkají mého zdraví.
4	I am constantly examining my health.	Průběžně si kontroluji své zdraví.
5	I am alert to changes in my health.	Změny zdravotního stavu mne znepokojují.
6	I am usually aware of my health.	Obvykle mám povědomí o svém zdraví.
7	I am aware of the state of my health as I go through the day.	V průběhu dne několikrát myslím na svůj zdravotní stav.
8	I notice how I feel physically as I go through the day.	Všímám si, jak se během dne cítím po tělesné stránce.
9	I am very involved with my health.	Svým zdravotním stavem se velmi zabývám.
